# A State-of-the-Art Review of FRP-Confined Steel-Reinforced Concrete (FCSRC) Structural Members

**DOI:** 10.3390/polym14040677

**Published:** 2022-02-10

**Authors:** Yu-Yi Ye, Jun-Jie Zeng, Pei-Lin Li

**Affiliations:** 1 School of Civil and Transportation Engineering, Guangdong University of Technology, Guangzhou 510006, China; yuyiyet@163.com (Y.-Y.Y.); 2112010031@mail2.gdut.edu.cn (P.-L.L.); 2 Department of Civil and Environmental Engineering, University of Macau, Macau 999078, China

**Keywords:** fiber-reinforced polymer (FRP) composites, hybrid systems, FRP-confined steel-reinforced concrete (FCSRC), structural strengthening/repair, buckling restrained braces (BRBs)

## Abstract

Fiber-reinforced polymer (FRP) composites have been widely used for strengthening or constructing structures due to their excellent corrosion resistance and high tensile strength. An emerging hybrid structural member form with FRP composites—which consist of a steel section as internal reinforcement, an external FRP wrap/tube, and concrete filled between them (referred to as FRP-confined steel-reinforced concrete (FCSRC) systems)—has attracted increasing research interest. To date, the concept has been adopted to strengthen/repair steel structures or used as new hybrid structural members (e.g., hybrid columns or beams, including buckling restrained braces (BRBs)). The FRP confinement and composite action between the three components in FCSRCs result in the excellent performance of the hybrid member. This paper presents a state-of-the-art review of FCSRCs for structural applications. The gaps in knowledge and future research opportunities on FCSRC structural members are also identified.

## 1. Introduction

Fiber-reinforced polymer (FRP) composites have been widely used as alternatives to steel reinforcement or strengthening materials in engineering structures due to their excellent corrosion resistance and tensile properties [1,2,3,4,5,6,7,8,9,10,11,12,13,14,15]. However, FRP composites also have many limitations including high costs, a low elastic modulus, and a lack of ductility. Therefore, it is generally not an economic option to construct pure FRP structures in practical applications. To this end, the combined usage of FRP composites and traditional construction materials (including steel and concrete) has attracted more and more attention in the research community [16,17,18,19,20,21,22,23,24,25,26,27,28,29,30,31], with the aim to establish cost-effective and novel forms of structures with excellent structural performance.

**Figure 1:**
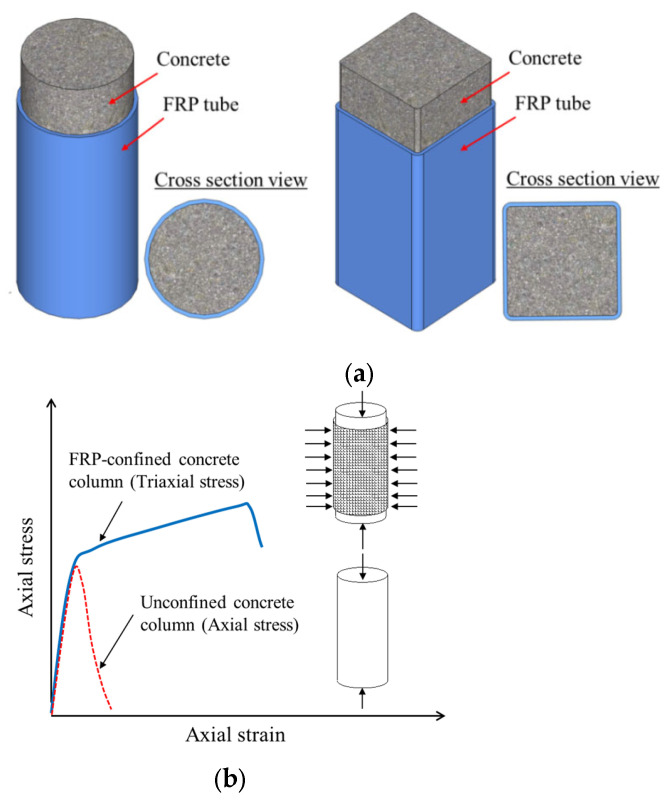
Concrete-filled FRP tubes (CFFTs). (**a**) Different shapes. (**b**) Performance improvement mechanism.

**Figure 2:**
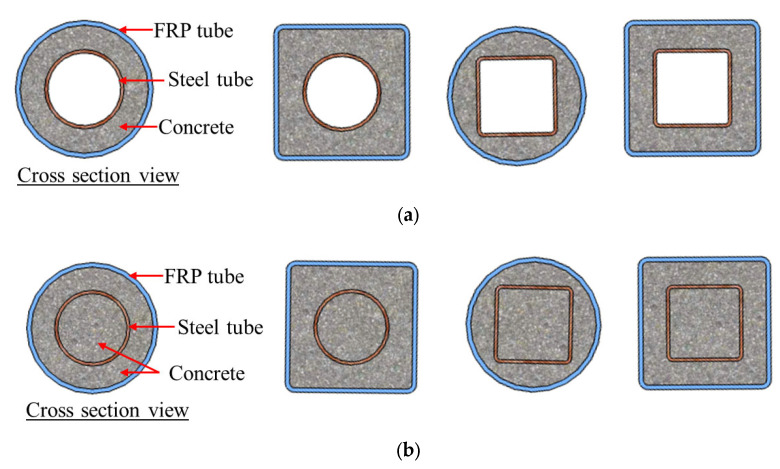
Double-skin tubular members (DSTMs) and double-tube concrete members (DTCMs). (**a**) Double-skin tubular members (DSTMs). (**b**) Double-tube concrete members (DTCMs).

**Figure 3:**
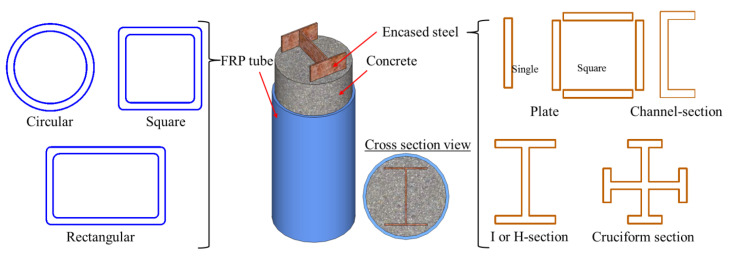
Different configurations in FRP-confined steel-reinforced concrete (FCSRC) members.

Among the various novel forms of hybrid structural members containing FRP composites, one of the most common is concrete-filled FRP tubes (CFFTs; Figure 1), in which the FRP tube provides passive confinement to the concrete core under compressive loading and further improves both the strength and deformation capacity of the concrete. Subsequently, capitalizing advantages of the FRP-confined concrete, steel reinforcement has been proposed to be used in FRP-confined concrete structural members to further enhance their deformation capacity and strength. Among these hybrid structural members, double-skin tubular members (DSTMs; see Figure 2a) [16,17,18,19,20] are popular. However, the DSTMs—which consist of an FRP tube as an external confining device and protective skin against environmental attacks, a steel tube as internal reinforcement, and concrete sandwiched between the two tubes—may experience the inward buckling of the steel tube under compression. To address this concern, some investigators have proposed filling the inner void of DSTMs to form the hybrid double-tube concrete members (DTCMs; see Figure 2b) [21,22,23,24,25,26,27]. Additionally, an emerging form of hybrid systems with FRP composites termed as FRP-confined steel-reinforced concrete structural members (FCSRCs; see Figure 3) [32,33,34,35,36,37,38,39,40,41,42,43,44,45,46,47,48,49,50,51,52,53,54,55,56,57]—which consist of a steel section as internal reinforcement, an external FRP confining tube, and concrete filled between them—has recently attracted increasing research interest.

The concept of FCSRC structural members was first proposed by Liu et al. [32] with the aim to strengthen/repair steel structures. Compared to conventional steel–concrete composite structural members or CFFT structural members, FCSRC structural members have the following advantages: (i) FRP wraps/tubes provide confinement to the concrete and prevent the possible buckling of the internal steel section so that both the concrete and the encased steel section substantially contribute to carrying the axial load; (ii) FRP tubes act as a stay-in-place formwork for the concrete and protect the steel and the concrete against corrosion; (iii) the steel section replaces the longitudinal steel reinforcement in traditional reinforced concrete (RC) members and provides additional shear and compressive capacities; and (iv) connections to the superstructures and foundations can be easily achieved due to the presence of a steel section in FCSRCs.

Up to date, FCSRC systems have been adopted to strengthen/repair steel structures [32,33,34,35,36,52,55] and as new hybrid structural members (e.g., hybrid columns, beams [37,38,39,40,41,42,43,44,45,46,47,48,49,50,51,53,54,56,57], or buckling restrained braces (BRBs) [58,59,60,61,62,63,64,65,66,67,68]). Extensive studies have been carried out to understand the performance of such forms of hybrid structural members, and they have been shown to have excellent performance. However, the current research related to hybrid systems has also not been carefully summarized, and further studies on FCSRC structural members are necessary for FCSRCs to be widely used in applications. To this end, this paper presents a state-of-the-art review of FCSRC structural members in strengthening steel structures/constructing new structures. The gaps in knowledge and future research opportunities on FCSRC structural members are also identified.

## 2. Development of FCSRC Structural Members

**Figure 4:**
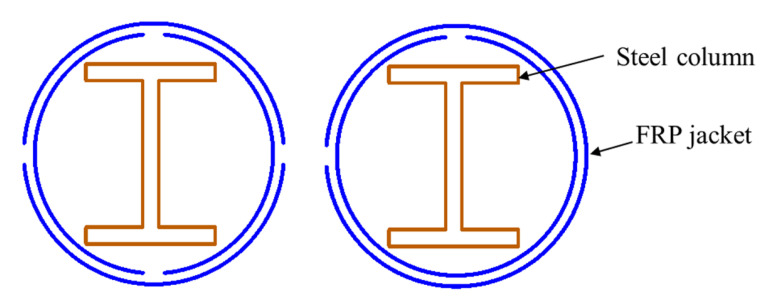
Fabrication schemes of FRP jackets.

**Figure 5:**
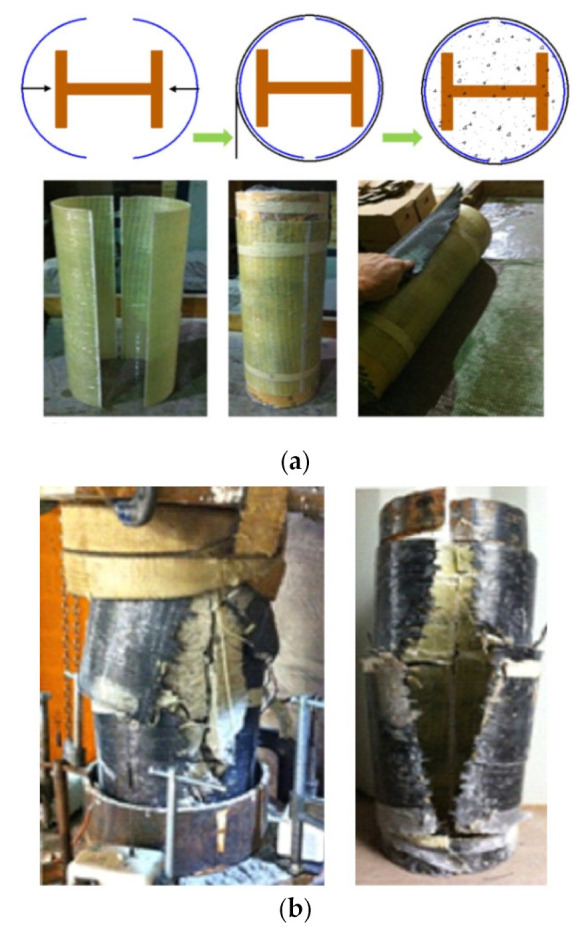
Fabrication schemes of FRP jackets (reproduced with permission from ref. [36], copyright American Society of Civil Engineers 2015 ). (**a**) Split-tube construction process. (**b**) Typical failure modes.

The concept of FCSRC structural members was first proposed by Liu et al. [32] to strengthen corroded I-shaped steel columns. In their study [32], a total of seven steel columns, in which five FCSRC specimens were notched to simulate the section loss caused by steel corrosion, were tested. The experimental results showed that the proposed FRP retrofitting technology for steel columns was feasible: the axial compression capacity of the reinforced steel column was significantly higher than that of the unreinforced steel column due to the confinement from the concrete and the FRP tube. In addition, as recommended by Liu et al. [32], the fabrication scheme of the FRP jacket was not limited to the FRP-wrapping-based wet layup process (i.e., FRP wraps). They can also be manufactured by bonding the separated FRP tubes together with epoxy to form an onion-skin jacket around existing steel columns (see Figure 4) in retrofitting applications, as placing the continuous FRP tube around an existing steel column is often difficult due to the interference of other structural elements. The capabilities of hybrid systems constructed with continuous and split FRP tubes are assumed to be similar provided that the bond strength between the epoxy and the FRP tube is efficient. Subsequently, the so-called onion-skin retrofitting technique was suggested to be applied to strengthen existing steel columns [33,36,37]. It should be mentioned that Karimi et al. [37] still utilized the continuous FRP tubes, and only Linde et al. [36] applied the typical retrofitting system to an existing I-shaped steel column. As shown in Figure 5a, a glass FRP (GFRP) tube was first cut into two half tubes, and then the two half tubes were brought together to surround an existing steel section. Next, carbon FRP (CFRP) sheets were wrapped around the split GFRP tube to create a continuous GFRP tube via a wet layup process. Finally, the concrete was cast into the void between the steel section and the FRP tube. Based on the experimental results, Linde et al. [36] concluded that the ultimate capacity of the composite columns could be significantly enhanced by using the proposed split-tube retrofitting technique, although the columns finally failed by FRP rupture at the gap in the GFRP tube that was externally wrapped with two plies of CFRP sheets (i.e., the weak point in the cross section) (see Figure 5b).

In addition to their use as a rehabilitation technique for steel structures, FCSRC systems have also gradually developed into significant structural elements in new structures (e.g., hybrid columns or beams). In such a hybrid structural member, the prefabricated FRP tube not only acts as a confining device but also serves as an in-site formwork for casting concrete. Moreover, both the confinement provided by the FRP tube and the support from the surrounding concrete can effectively restrain local and overall buckling of the internal steel section, leading to the full exploitation of the load-carrying capacity of the steel section. In general, structural capabilities are similar whether FCSRC systems are used for structural repairing/strengthening or new construction. Currently, a number of experimental and theoretical investigations have been conducted to gain an in-depth understanding of the behavior of this novel form of hybrid member under various loadings (e.g., concentric compression, eccentric compression, and bending) [32,33,34,35,36,37,38,39,40,41,42,43,44,45,46,47,48,49,50,51,52,53,54,55,56,57]. This paper summarizes the experimental research associated with FCSRC systems in strengthening/repairing steel columns or constructing new structures (Table 1). As presented in Table 1, previous studies have mainly focused on the behavior of FCSRC columns subjected to concentric or eccentric compression. The investigated parameters varied and included the sectional configuration, the type of the three constituent materials, the slenderness ratio, and the load eccentricity.

**Figure 6:**
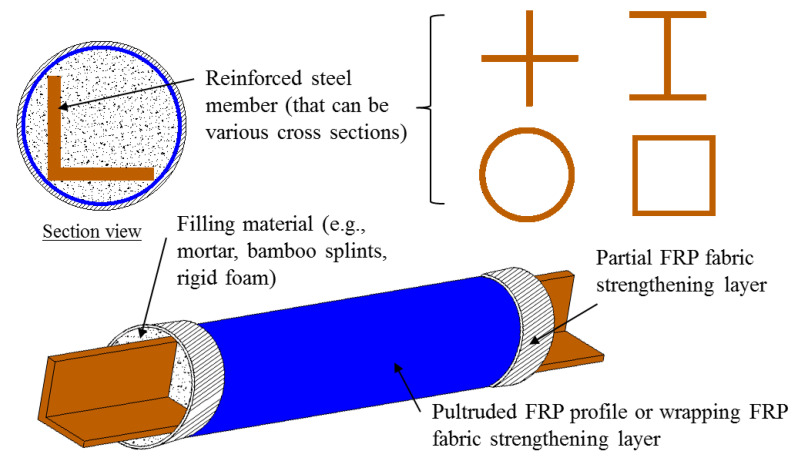
Different configurations in FCSRC BRBs.

On the other hand, the concept of FCSRC systems has been applied to the buckling restrained braces (BRBs) that are commonly used as passive energy dissipation devices in seismic zones (i.e., stabilizing steel braces with FRP reinforcements) [58,59,60,61,62,63,64,65,66,67,68]. As shown in Figure 6, in such a form of BRBs, FRP composites are often used as the external shell, which can provide confinement to improve the buckling behavior and ultimate strength of the steel core. Additionally, the filling materials in this form are not limited to concrete; contrarily, they are often replaced by other light-weight materials (such as self-consolidating grout, cement mortar, or bamboos splints) that are intended to restrain the buckling of the steel core not resisting the applied loads. Moreover, lubricants or isolation materials are often used to eliminate the bond and friction between the steel core and the filling materials, as well as the bond between the filling materials and the external FRP wrap/tube [62,64]. The goals are to ensure ductile failure of the brace and to avoid the cracking of the filling materials subjected to loadings, which would weaken the lateral buckling support. However, some researchers have allowed the fillers to bond directly to the steel core [59]. Some attempts have been made to verify the feasibility of FCSRC systems in BRB applications. Table 2 provides a summary of studies on FCSRC BRBs, and the following sections present a comprehensive review of the relevant works of BRBs with FCSRC systems.

## 3. Applications in Strengthening Existing Structures/Constructing New Structures

### 3.1. Different Configurations

As shown in Figure 3, both the steel and the FRP wrap/tube in FCSRCs can be designed with different cross-sectional shapes. Generally, different cross-sectional shapes of the two components (i.e., the steel and the FRP wrap/tube) have different effects on the structural performance of FCSRCs. For instance, circular wrap/tube can provide more sufficient confinement to concrete than the square or rectangular wrap/tube. To this end, this section comprehensively reviews the latest innovative studies of FCSRCs with different configurations, providing reference for follow-up research and practical applications.

#### 3.1.1. Cross Section Shape

In building construction, square or rectangular columns may be preferred to circular columns due to aesthetical and other reasons. However, it is well-known that not all the concrete in a square or rectangular FRP tube is effectively confined, especially the concrete close to the flat sides. This hinders the application scope of FRP-confined concrete columns with a non-circular cross section. As shown in Table 1, most studies on FCSRC columns have focused on circular columns. The structural performance of the non-circular FCSRC columns with profile steel has also been explored [34,35,39,42,44,45,46,50]. Generally, circular FCSRC columns are superior to non-circular FCSRC columns in terms of the load-carrying capacity if other parameters are identical and the difference between them is attributed to the different confinement mechanisms of the concrete in circular and non-circular FRP tubes.

#### 3.1.2. Steel Shape

##### I- or H-Section Steel

**Figure 7:**
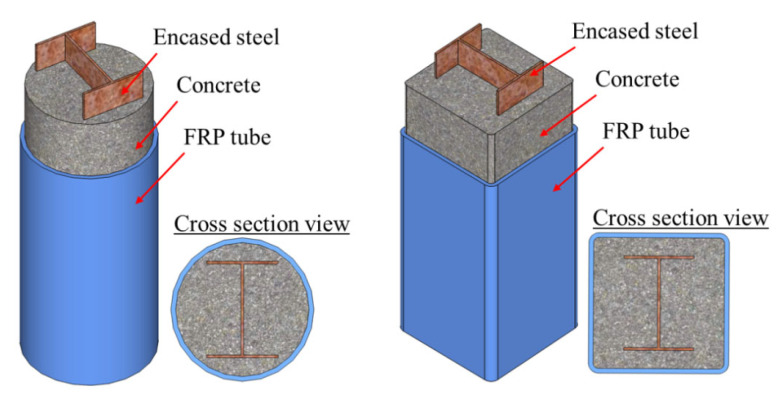
FCSRC columns with I or H-section steel.

**Figure 8:**
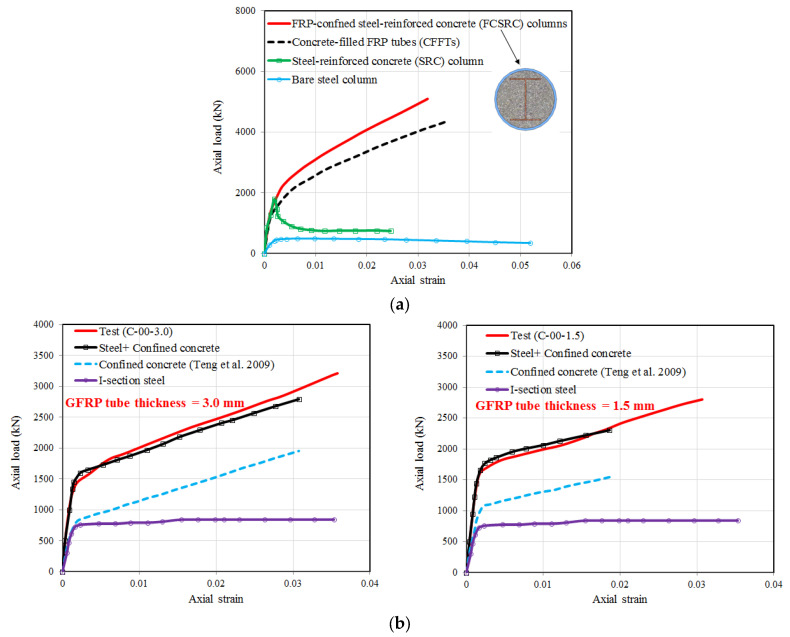
Axial load–axial strain curves of FCSRC columns with I-section steel under concentric compression (reproduced with permission from Chen et al. [41] and Yu et al. [50], published by Eng. Struct. 2020, 220, 110990, and Compos. Struct. 2016, 154, 493–506, respectively). (**a**) Chen et al. [41]; (**b**) Yu et al. [50].

I-shaped (or H-shaped) steel sections are one of the most common forms in steel structures, and they have recently been used in FCSRC columns (see Figure 7) [32,34,35,37,38,39,40,41,43,46,47,48,50,52,53,55,56]. Previous studies on FRP-confined concrete-encased I-section steel columns have indicated their excellent ductility under various loading scenarios (e.g., concentric compression and eccentric compression). In such a sectional configuration, the buckling of I-section steel (especially overall buckling) can be restrained so that the post-yield strength of the steel can be fully exploited and the concrete is effectively confined by the external FRP confining device. Most importantly, the I-section steel may suppress the lateral expansion of the in-filled concrete because the two flanges are connected by the web and its confinement level is depended on the flexural stiffness of the flanges and the axial stiffness of the web [50]. This explains why both the load-carrying capacity and deformation capacity of FCSRC columns are superior to those of bare steel columns and steel-reinforced concrete columns (see Figure 8a). Yu et al. [50] reported that a combination of the three constituent materials (i.e., FRP, concrete, and steel) in this form of hybrid system achieved beneficial interactions between them (see Figure 8b). In addition, longitudinal reinforcing bars have sometimes been embedded to compensate for the loss of the weak-axis bending stiffness and increase the capacity of corroded and buckled steel columns [52,55].

**Figure 9:**
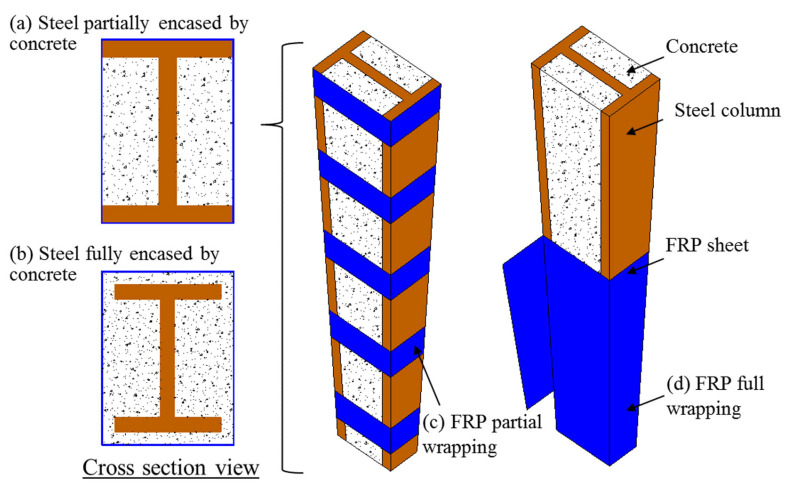
Schematic of composite columns. (**a**) Steel partially encased by concrete; (**b**) Steel fully encased by concrete; (**c**) FRP partial wrapping; (**d**) FRP full wrapping.

In most cases, I- or H-shaped steel columns are fully encased by the concrete and the composite columns are fully confined by an FRP jacket along with the column height [32,36,37,38,39,40,41,43,47,48,50,52,53,55,56]. On the other hand, some scholars have also investigated the partial encasement mode of the steel section and different strengthening techniques of the FRP jacket in FCSRCs with I-section steel (see Figure 9) [34,35,46]. For instance, in the studies of Karimi et al. [34,35], the steel section in the rectangular columns was partially encased by concrete and the entire composite column section was fully wrapped with an FRP jacket (see Figure 9d). They found that the compressive behavior (e.g., strength, elastic axial stiffness, and deformation capacity) of the partially encased steel columns could be significantly enhanced with the increase in either the thickness or the corner radius of the FRP tube. However, no studies have reported differences in full and partial encasement modes for I- or H-section steel in FCSRC columns to date. The flanges of the steel section that are not effectively supported by concrete may be more prone to buckling than that of the fully encased steel section, leading to the premature rupture of the FRP jacket. Note that Karimi et al. [37,38] adopted the prefabricated FRP tube instead of the FRP wrap. They also accounted for the load-carrying capacity of the FRP tube (as elastic materials) when evaluating the ultimate strength of the composite columns. Liang et al. [46] adopted the same strengthening device as Karimi et al. [34,35] for steel columns (i.e., partial encasement mode), but they adopted FRP partial wrapping strengthening schemes for entire composite columns (see Figure 9c). They conducted a series of experimental studies to understand the compressive behavior of CFRP partially wrapped steel-reinforced concrete stub or slender columns. The results showed that CFRP strips could improve the ultimate strength and stiffness of partially concrete-encased steel columns and effectively delay the local buckling of profile steel. These results indicate the feasibility of using CFRP strips to confine partially concrete-encased steel columns.

##### Cruciform Section Steel

**Figure 10:**
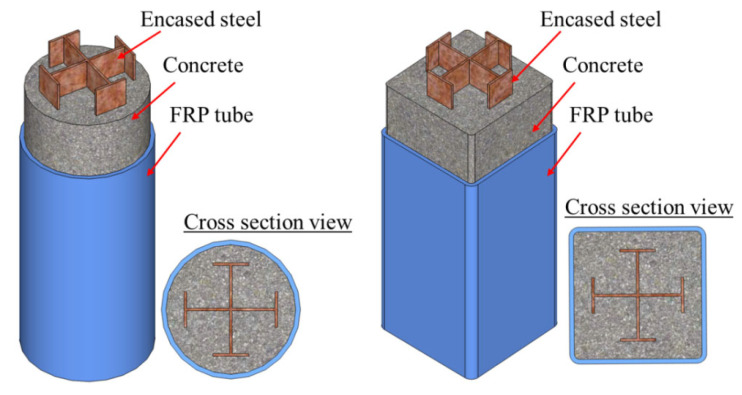
FCSRC columns with cruciform section steel.

Studies on FCSRC columns with a cruciform steel section have been limited (see Figure 10) [41,42,44,45,47,49]. This form of composite columns was first proposed by Huang et al. [42]. Compared to I-shaped steel, the cruciform section steel with two pairs of flanges connected by the webs provides a more effective confinement to the concrete, which can compensate for the insufficient confinement from the FRP of the concrete near the four flat sides of a square section. The cross-shaped steel section is also particularly advantageous for columns carrying loads in two lateral directions. It should be pointed out the studies of Huang et al. [42,44,45] focused on square FCSRC columns, where the cruciform section steel was partially encased by the concrete (i.e., there was no concrete cover between the steel flange and the FRP tube). In addition, Huang et al. [42,44,45] investigated the effects of the flange width, flange thickness, web thickness, and FRP tube thickness on the compressive behaviors of FCSRC columns via experimentation. Similar studies have also been conducted by Chen et al. [41], Ren et al. [47], and Xiong et al. [49] on the compressive behavior of circular FCSRC columns. The test results from Huang et al. [42] confirmed the superior performance of FCSRC columns with cruciform steel: the buckling of the steel section was effectively prevented (which was consistent with the findings of Ren et al. [47]), contributing to a ductile response of the composite column, and both the compressive strength and ultimate axial strain of the confined concrete in square FCSRC columns were superior to those in circular CFFTs with an identical internal cross section and the same FRP tube. That is, the axial strength of an FCSRC column was larger than that of the sum of the strengths of the CFFT column and the cruciform section steel column due to the additional confinement and stability of the steel columns. Moreover, Ren et al. [47] reported that the load capacity of FCSRC columns was very close to the summed loads of a CFFT column and a steel column without buckling behavior.

##### Channel Steel and Steel Plate

**Figure 11:**
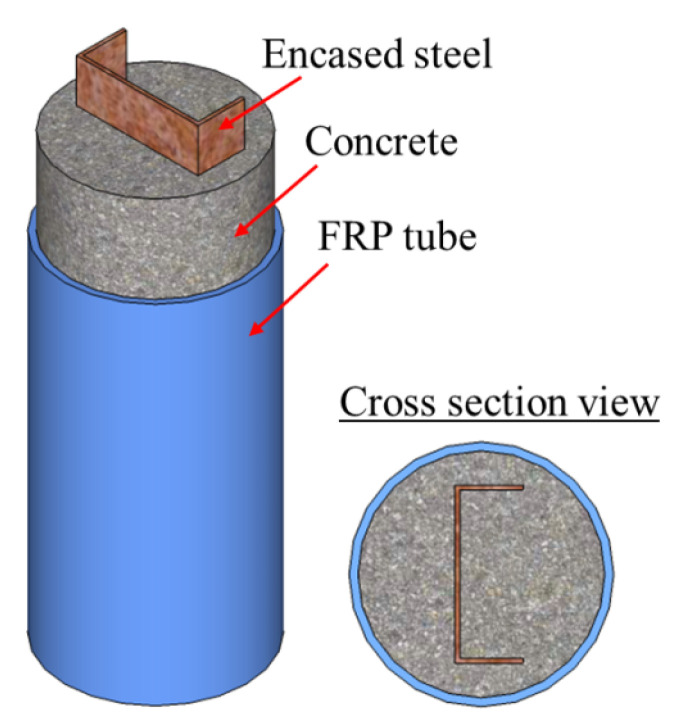
FCSRC columns with channel steel.

**Figure 12:**
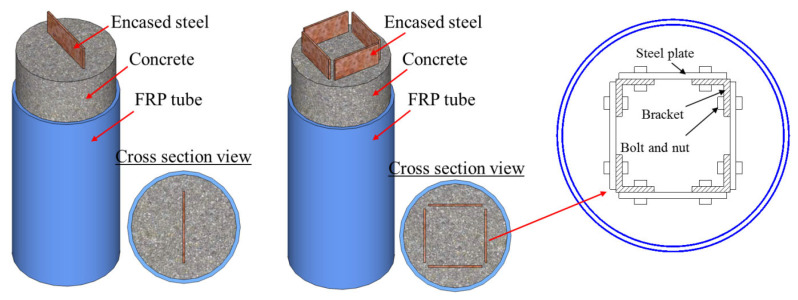
FCSRCs with a single steel plate or multiple steel plates.

In comparison with I-shaped steels and cruciform section steels, channel steels and steel plates are less popular in FCSRC columns due to the asymmetry of their cross sections and other reasons. To the authors’ best knowledge, only He and Chen [54] and Yu et al. [51] have conducted experimental studies on the compressive behavior of FCSRC columns with a channel steel section (Figure 11) or a steel plate (Figure 12), respectively. He and Chen [54] investigated the effects of concrete strength grade, the steel ratio, and the diameter-to-thickness ratio of the GFRP tube. They found that in addition to the ultimate load capacity and initial stiffness, the deformation capacity of the composite column increased with the concrete strength, but Xie et al. [56] reported that I-section steel-reinforced CFFT specimens with a higher concrete strength had a lower deformation capacity. Moreover, the variation in the steel ratios had a negligible effect on the deformation of the FCSRC column with channel steel [54], which was consistent with the findings of Xie et al. [56].

In addition, in the study of Yu et al. [51], encased high-strength steel plates were connected by bolted angle brackets at discrete heights (see Figure 12). The novelty of the proposed method is that welding was not involved in the fabrication of high-strength steel profiles, thus reducing the manufacturing and transportation costs and avoiding the perceived difficulties associated with welding high-strength steel sections, leading to a simpler structural design procedure. The test results from Yu et al. [51] demonstrated that the buckling of steel plates with a yield stress of 455 MPa could be well-prevented by the encasing concrete before and after the rupture of the FRP tube, and the FCSRC columns possessed a good ductile responses under concentric and eccentric loadings. Moreover, no buckling of the steel plate was observed in the FCSRC columns with a single plate after the respective FRP tube ruptured at a large deformation, so they concluded that the occurrence of buckling of the square plates before the rupture of the FRP tube may have been affected by the thickness of the concrete cover, though this has not been verified to date.

### 3.2. New Types of Materials Used in FCSRC Columns

#### 3.2.1. FCSRC Columns with LRS FRP Tube

FRP composites can be classified into two types according to their tensile strain capacity: large rupture strain (LRS) FRPs (up to 8%) and small rupture strain FRPs (i.e., conventional FRPs). Conventional FRPs include CFRPs, GFRPs, aramid FRPs (AFRPs), and basalt FRPs (BFRPs). Generally, CFRPs have the highest ultimate strength and modulus of elasticity, while BFRPs have the lowest ultimate strength and modulus of elasticity, as shown in Table 3. In terms of strain capacity, CFRPs generally have a lower ultimate strain than BFRPs and GFRPs. On the other hand, the fibers utilized in LRS FRPs include PA (polyacetal), PEN (polyethylene naphthalate), and PET (polyethylene terephthalate) fibers, and the respective composites are referred to as PA FRPs, PEN FRPs, and PET FRPs, respectively. Slightly different from conventional FRPs, LRS FRPs possess a large rupture strain and a much lower modulus of elasticity. In particular, LRS FRPs exhibit an approximately bilinear tensile stress–strain behavior, while conventional FRPs are linear-elastic materials. As shown in Table 1, recent studies have mainly focused on FCSRC columns using GFRPs and CFRPs. Huang et al. [43] investigated FCSRC columns with PET FRP tubes. The results showed that the axial deformation capacity of PET FCSRC columns (an ultimate axial strain of above 0.075) was much better than that of FCSRC columns with a GFRP tube (an ultimate axial strain of around 0.012). Although the columns had experienced sustainable deformation, the local buckling of the embedded I-shaped steel was still restrained by the surrounding confined concrete and the steel section may provide additional confinement, results consistent with the findings of Huang et al. [42].

#### 3.2.2. FCSRC Columns with High-Strength Steel

High-strength materials are often preferred when the weight and/or size of structures needs to be reduced. However, the use of high-strength materials has so far been rather limited because they significantly reduce the ductility of members. High-strength steel (generally refers to the steel with a yield strength greater than or equal to 450 MPa) is more susceptible to buckling failure than normal-strength steel. Such buckling failure should be avoided in structures because it portends that the yield strength of high-strength steel cannot be fully utilized and the ductility of the structural member can be greatly compromised. Efforts have been made to prevent or delay the local and overall buckling of steel in structural columns, such as (i) filling a steel tube with concrete [69,70,71], (ii) filling the annular space between the steel tube and the FRP tube for double-skin tubular columns (DSTCs) [16,17,18,19,20], (iii) implementing an FRP wrap on the concrete-filled steel tubes [72,73,74,75,76], and iv) encasing a steel section (e.g., tube shape or I-section) by concrete [77,78,79,80] or FRP-confined concrete [21,22,23,24,25,26,27,32,33,34,35,36,37,38,39,40,41,42,43,44,45,46,47,48,49,50,51,52,53,54,55,56,57]. However, each technique may have limitations: (i) for the first one, the local outward buckling of the steel tube still occurs due to incompatibility with concrete (the Poisson’s ratios of concrete and steel are 0.18 and 0.3, respectively); (ii) for the second one, the possible inward buckling of the steel tube remains a potential issue for DSTCs; and (iii) for the third and fourth ones, the steel section may be less likely to buckle prior to the cracking of concrete (or the FRP tube rupture) because it is surrounded by the concrete or constrained by the FRP jacket. Recently, Yu et al. [51] adopted a high-strength steel section in FCSRC columns (i.e., corresponding to the fourth one) (Figure 12). It was demonstrated that the buckling of steel plates with a yield stress of 455 MPa could be well-prevented by the encasing concrete and the plates’ yield strength could be fully utilized in FCSRC columns, leading to excellent structural responses. Therefore, high-strength steel is recommended for FCSRC columns, though further experimental works are still needed to validate its feasibility in the near future.

#### 3.2.3. FCSRC Columns with New Types of Concrete

In recent years, scholars have extended the in-filled concrete in FCSRC columns from ordinary concrete to new types of concrete (e.g., high-strength concrete, recycled aggregate concrete, and expansive concrete), which has considerably expanded the application scope of FCSRC columns. The following sections summarize the latest studies on FCSRC columns with new types of concrete.

##### High-Strength Concrete

High-strength concrete (with a compressive strength of 50–120 MPa) has become increasingly attractive due to its high strength and high modulus of elasticity. Previous studies have revealed that FRP confinement can improve both the strength and ductility of high-strength concrete; however, a strain-softening segment often appears in the stress–strain behavior of FRP-confined high-strength concrete. The interaction between the encased steel and the high-strength concrete confined with FRP is also an important issue to be explored for FCSRC columns. Up to date, there have been few studies on the compressive behavior of FCSRC columns using high-strength concrete [41,53]. In the study of Ozbakkaloglu and Fanggi [53], two FCSRC columns with high-strength concrete of 102.9 MPa were tested just as reference specimens for DSTCs. It was revealed that the concrete in FCSRC columns exhibited a slightly lower strength than that of the DSTCs, indicating good confinement from the external FRP confining tube. In the study of Chen et al. [41], four FCSRC columns with high-strength concrete of around 110 MPa were tested. It was demonstrated that the high-strength concrete produced a higher axial stiffness and a higher axial compressive capacity for the FCSRC columns than the normal-strength concrete, but it weakened the deformation capacity of the composite column. These findings were consistent with those of previous studies [14,15,16,17,18,19,20,21,22,23,24,25,26,27].

##### Recycled Aggregate Concrete

Extensive research have been carried out to understand the material and structural performance of recycled aggregate concrete [81,82,83,84], and some design methods have been proposed for structural members with recycled aggregate concrete [85,86]. However, the use of recycled aggregate concrete is still limited to non-structural elements due to its inherent drawbacks (e.g., higher water absorption, weaker interfacial transition zones, lower strength, and lower stiffness). Notwithstanding, it has been proven that both the strength and deformation capacity of recycled aggregate concrete can be improved by FRP confinement [84]. Recently, some researchers used recycled aggregate concrete in FCSRC columns [48,49]. Xiong et al. [49] explored the effects of the replacement ratio of recycled coarse aggregates and the FRP confining stiffness on circular FCSRC columns with a cruciform steel section. It was found that FCSRC columns with recycled aggregate concrete had similar compressive behavior to those with ordinary concrete, but the use of recycled aggregate concrete slightly decreased the load-carrying capacity of the column and led to a low concrete dilation at a certain axial deformation in the strain-hardening segment. In contrast to Xiong et al. [49], Ren et al. [48] applied recycled aggregate concrete to a circular FCSRC column with an I-shaped steel section and focused on the behavior of the slender columns under eccentric compression. They found that with the exception of the ultimate load-carrying capacity, the buckling behavior of slender FCSRC columns was hardly affected by the replacement ratio of recycled aggregate concrete. Additionally, the load-descending rate tended slow as the replacement ratio increased.

##### Expansive Concrete

It is well-known that FRPs provide passive confinement to concrete cores subjected to lateral dilation [12,13,14,15]. Thus, concrete shrinkage and stress hysteresis could weaken the utilization ratio of FRP confinement. In this case, it is recommended to use expansive concrete to provide a small amount of active confinement [87,88,89]. Recently, several researchers adopted expansive concrete in FCSRC columns to generate active pre-stress for strengthened steel cores [32,36,37,39,40,52]. Liu et al. [32] first proposed to use expansive light-weight concrete as the filling materials of wrapped steel columns. Their results revealed that the composite columns using expansive concrete-generated pre-stress had an increase in the ultimate load capacity compared to the control specimens (i.e., composite columns made with non-expansive light-weight concrete). Subsequently, Karimi et al. [37] and Linde et al. [36] undertook experimental investigations to explore the effect of concrete shrinkage on confined concrete and composite columns. It was found that the addition of a shrinkage reduction agent had a significant effect on the confined concrete strength and the compressive behavior of composite columns was greatly improved. Cao et al. [39,40] incorporated expansive concrete in the square and circular FCSRC columns, and they found that the pre-stress generated from the expansive concrete could eliminate the stress lag and that the strength of expansive concrete-based FCSRC columns was higher than that of ordinary concrete specimens.

### 3.3. Behavior of Slender FCSRC Columns

Different from stub FRP-confined composite columns, slender FRP-confined composite columns encounter secondary bending moments, which reduce their load-carrying capacity. This is because the increased slenderness changes the failure mode of the column from a loss of cross-sectional strength to a loss of member stability, thus leading to a reduction in the FRP confinement efficiency. Therefore, the effect of column slenderness on the compressive behavior of columns is an important issue to be addressed. To the best knowledge of the authors, most of the research on FCSRC columns has focused on stub columns, and studies on slender FCSRC columns are limited [35,38,44,46,48,50]. The following sections review the state-of-the-art studies of the buckling behavior of slender FCSRC columns under concentric or eccentric loadings.

#### 3.3.1. Concentrically Loaded FCSRC Columns

Karimi et al. [35,38] investigated the influence of the slenderness ratio on the behavior of rectangular FCSRC columns. As expected, the load-carrying capacity of the composite columns and the beneficial effect from the FRP confinement both decreased with an increase in the slenderness ratio. A similar finding was also reported in the study of Liang et al. [46]. In particular, as observed by Karimi et al. [35,38], the FRP confinement was invalid in composite columns with a slenderness parameter (λ) greater than 1.0 due to the elastic overall buckling of the column prior to confinement activation. However, as these studies were aimed at strengthening the damaged steel columns, the performance of the long composite columns (e.g., the strength, the axial stiffness, and the energy capacity) was still significantly enhanced compared to the bare long steel columns.

#### 3.3.2. Eccentrically Loaded FCSRC Columns

In practical applications, columns are inevitably subjected to combined compression and bending. The load-carrying capacity of a column is generally reduced by a bending moment due to uneven stress distribution. Huang et al. [44] investigated the effects of the slenderness ratio and load eccentricity on the compressive behavior of slender FRP-confined concrete-encased cross-shaped steel columns. The results indicated that the load-carrying capacity of FCSRC columns decreased with the slenderness ratio and the load eccentricity, as expected; however, the lateral deformation at the ultimate state (i.e., at the time of FRP rupture) increased with the slenderness or the load eccentricity. Moreover, Ren et al. [48] reported that slender FCSRC columns under eccentric loadings experienced different failure modes in compression zones compared to short FCSRC columns. For the former, the failure was governed by matrix rupture, while the failure modes of the latter varied from hoop rupture to matrix rupture with the increase in eccentricity.

### 3.4. Theoretical Models

Models for the load capacity and ultimate axial strain of FCSRC columns/respective confined concrete are summarized in Table 4. These models are classified into three types (i.e., Type I, Type II, and Type III) based on the estimation of the contributions of three different components (i.e., FRP, concrete, and steel, respectively) in FCSRC columns. The contributions of three different components are generally considered in separation; the following assumptions are adopted in those theoretical models: (i) the steel was idealized as an elastic-perfectly plastic material; (ii) for in-filled concrete, only the FRP confinement (and even the active confinement from expansive concrete) was considered while the additional confinement from the steel section was neglected, so the behavior of in-filled concrete was often assumed to be similar to that of the concrete confined with the FRP; and (iii) the longitudinal contribution of the FRP tube was considered in some studies [37,47,56,90].

‘Type I’ considers the contributions of unconfined concrete and profile steel in the nominal load capacity $P_{co}$ of FCSRC columns [39]. As the external FRP shell plays a good role in confining the inner steel and the filling concrete, the ultimate load capacity of FCSRC columns is often larger than the nominal load capacity, and the latter is conservative in design. To this end, Cao et al. [39] proposed theoretical models for the compressive strength and the ultimate axial strain of confined concrete in FCSRC columns, which accounted for passive and active confinements from the FRP and expansive concrete, respectively. It was revealed that the model had a higher accuracy in predicting compressive strength than ultimate axial strain (i.e., overestimating the test results).

‘Type II’ considers the contributions of confined concrete and profile steel in the load capacity $P_{cc}$ of FCSRC columns [41,45,46,48,49,54]. It should be mentioned that Chen et al. [41] and Xiong et al. [49] calculated the superimposed load capacity $P_{o}$ in assessing the composite effect between the three components in FCSRC columns. It was interesting to find that the composite effect of FCSRC columns with cruciform section steel was not enhanced by combining CFFT and steel columns due to the local buckling of the steel, but the composite effect existed in FCSRC columns with I-shaped steel because the local buckling such steel did not occur. Moreover, Liang et al. [46] added a contribution from the partial confinement of FRP strips to the design equation of Eurocode 4 [91], which was originally proposed for predicting the load capacity of steel-reinforced concrete columns; the predicted results were shown to be in good agreement with their own test results. Different from Liang et al. [46], the composite columns in the study of Huang et al. [45] were fully wrapped by FRP sheets, so the authors adopted the model of Teng et al. [92] to predict the ultimate axial strength and corresponding axial strain of the confined concrete in FCSRC columns. It was reported that the proposed method showed reasonable agreement with the test load capacity of FCSRCs but relatively conservative predictions of the ultimate axial strain of FCSRCs. He and Chen [54] proposed a confinement coefficient θ to consider the effect of three parameters (i.e., the steel ratio, the concrete compressive strength, and the diameter–thickness ratio of the GFRP tube) on the enhancement of the axial load capacity of the composite column. However, the design formulas have been limited to the test results of the proposers of the models, so they cannot capture various FRP confinement levels, which are key factors for confined concrete columns. Last but not least, Ren et al. [48] proposed a design calculation model to determine the ultimate load capacity of the slender FCSRC columns under eccentric compression. This model accounted for the effects of slenderness and eccentricity, and the ultimate axial strength model of FRP-confined concrete was based on the model of Teng et al. [92].

**Figure 13:**
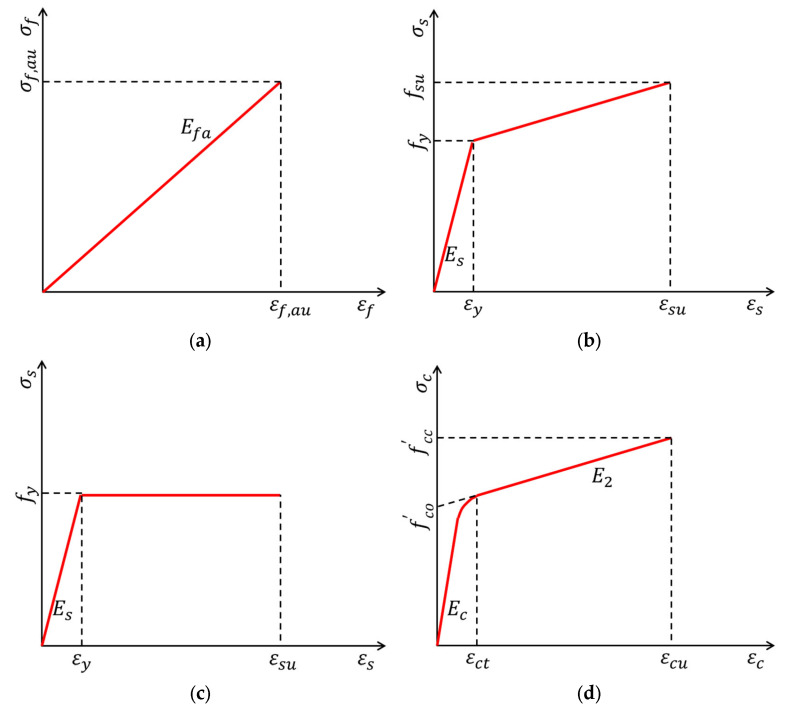
Stress–strain relationships for FRP tube, steel, and confined concrete. (**a**) FRP tube. (**b**) Steel (Model I). (**c**) Steel (Model II). (**d**) Confined concrete.

‘Type III’ considers the contributions of three parts (i.e., confined concrete, profile steel, and FRP tube) in the load capacity $P_{cc}$ of FCSRC columns [37,47,56,90]. In the studies of Karimi et al. [37,90] and Ren et al. [47], prefabricated FRP tubes were approximated by orthotropic elastic membranes and the proposed models accounted for the biaxial behavior of the FRP tubes (Figure 13a), while Xie et al. [56] directly adopted the compressive strength of a hollow FRP tube. Note that in the model of Karimi et al. [37,90], the steel was assumed to present an elastic–plastic response with a strain hardening from the yield stress $f_{y}$ to the ultimate stress $f_{su}$ (Figure 13b), which was different from the assumptions in other studies (e.g., steel has a yield platform; see Figure 13c). Furthermore, Karimi et al. [37,90] directly adopted the model of Lam and Teng [93] (Figure 13d) to predict the compressive strength of confined concrete, and Ren et al. [47] adopted a stress-path-dependent, passively confined concrete stress–strain model to predict the ultimate axial strength of confined concrete in FCSRC columns. Moreover, in the model of Xie et al. [56], the strength of the composite section (i.e., steel and concrete) was represented by a conversion strength, and the model considered the beneficial effect from the FRP confinement on the enhancement of the conversion strength. In addition to the strength model of the composite column, Karimi et al. [90] also developed an analytical model to predict the axial compressive behavior of composite columns for various slenderness ratio values. It should be noted that these four models have not been verified by test results of other studies despite providing close predictions with their own test results.

### 3.5. Flexural Behavior of FCSRC Beams

**Figure 14:**
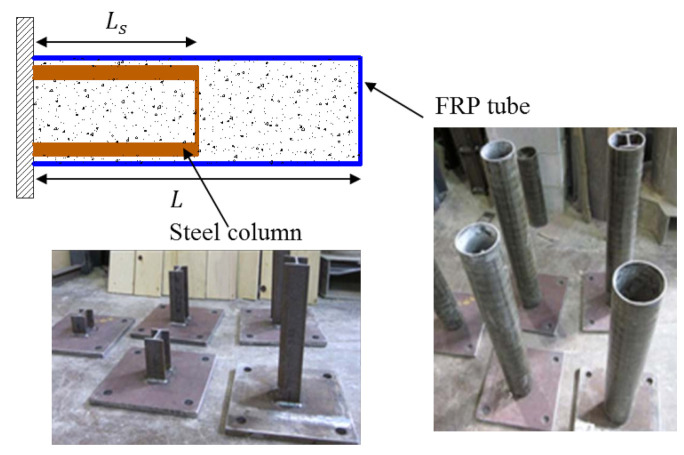
Specimens in cantilever bending tests (reproduced with permission from ref. [57], copyright American Society of Civil Engineers 2012).

Studies on the flexural behavior of FCSRC beams have been rather limited. Up to date, only Zakaib and Fam [57] have investigated the flexural performance of circular concrete-filled GFRP tube-encased I-section steel beams via experimentation. A total of ten beam specimens, including steel and CFFT control specimens, was tested under four-point bending, with the key parameters being the beam diameter, the GFRP tube thickness, and the laminate structure of the GFRP tube. It was revealed that the FCSRC beams with fiber angles of [−84/+6] had considerable increases in flexural strength, stiffness, and pseudo-ductility, which was attributed to the presence of the I-section steel. However, for FCSRC beams with fiber angles of [+54/−56], ductility was not improved by the presence of I-section steel because the CFFTs with fiber angles of [+54/−56] inherently possessed a ductile response. In addition, they addressed a moment connection through five cantilever bending tests (Figure 14) in which the embedded I-section steel was welded to a steel base plate, and they proposed a model to predict the moment capacity of the connection. They found that the strength and ductility of this connection primarily depended on the depth of the steel section embedded in the CFFT member. The minimum embedded lengths of the steel section required to reach the flexural strength of the CFFT member and the full plastic capacity of the moment connection at the fixed end were 17% and 48% of the CFFT span, respectively.

## 4. Application in Buckling Restrained Braces (BRBs)

Recently, FRP composites with high stiffness have been adopted to prevent the local and overall buckling failure of slender steel members [58,96,97,98]. Many efforts have been made to apply the concept of FCSRC systems in BRBs for structural repairing [58,59,60,61,62,63,64,65,66,67,68]. As shown in Table 2, previous studies on FCSRC BRBs have focused on behavior under axial compression or reversed cyclic loading. The studied parameters included variables of the constituent materials and the bond properties between them. Although the filling materials varied from study to study, BRBs can be divided into two categories: pre-fabricated BRBs and assembled BRBs. The former are usually made of pultruded FRP tubes, and the latter are made of FRP wraps. To this end, this section reviews the latest innovative research of FCSRC BRBs from these two aspects.

### 4.1. Pre-Fabricated BRBs

**Figure 15:**
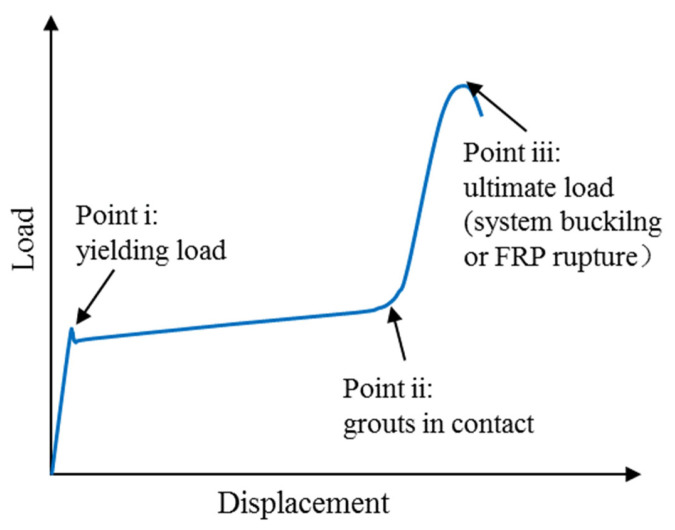
Typical load–displacement curve of BRBs (adapted from MacEachern and Sadeghian [62]).

MacEachern and Sadeghian [62] tested 27 hybrid BRBs with a hot-rolled rectangular steel bar as core, self-consolidating grout as fillers and external GFRP tubes. They found that the hybrid BRBs could be designed to change the failure modes of the steel core from a sudden buckling failure to a ductile yielding when sized correctly. A typical load–stroke curve is presented in Figure 15, in which the response is divided into three segments: (i) Point i is the first peak load corresponding to the yielding (or bulking) of the steel core; (ii) point ii is the point at which the loading platen begins to contact the entire section and the grout carries the load; and (iii) point iii is the ultimate failure load corresponding to the overall system buckling or FRP shell rupture. They also revealed that increasing grout strength did not significantly improve the flexural rigidity for BRBs with smaller diameters, and the influence of the number of FRP confining layers on the overall flexural rigidity of the system was relatively small, especially for BRBs with larger diameters.

Similarly, Feng et al. [58] demonstrated the feasibility of FRP strengthening technique on BRBs via experimentation on L-shaped steel members with a certain slenderness, filled with bamboo splits or high-strength, non-shrinkage grout and externally confined with pultruded GFRP tubes. Subsequently, Feng et al. [59] conducted more thorough research on BRBs with mortar-filled pultruded GFRP tubes with different bi-axial symmetrical cross sections (cross/I/round/square) by experimental and theoretical modelling. It was concluded that the use of more FRP fabric in the middle of the brace (more than two layers) was redundant because it contributed little to the load carrying capacity and ductility, which was consistent with the findings of MacEachern and Sadeghian [62]. Three typical failure modes of strengthened specimens at peak load were also summarized in the study of Feng et al. [59].

Feng et al. [61] built finite element analysis (FEA) models to continually study more parameters (yield and ultimate strength of the steel, un-strengthened steel member length, interfacial bond, and initial imperfection) that affect the compressive behavior of BRBs with mortar-filled FRP tubes under axial compression. It was revealed that the ultimate load capacity of BRBs could increase with increases in the yield and ultimate strength of the steel and the bond strength of interface but decrease with increases in un-strengthened steel member length and initial imperfection.

Several studies have focused on the cyclic performance of BRBs. Sun et al. [63] investigated the hysteretic behavior of the pre-fabricated BRBs by testing seven BRB specimens with concrete-filled GFRP tubes and three BRB specimens with concrete-filled steel tubes. They found that global buckling only occurred in the medium- and long-length BRBs confined with filament-wound GFRP tubes (fibers in the hoop direction with a winding angle of 56°), while BRBs confined with pultruded GFRP tubes (fibers in both hoop and longitudinal directions) exhibited more stable hysteretic responses in which the maximum compressive loads could exceed about $1.4f_{y}A$ and no global buckling occurred. Thus, Sun et al. [63] suggested the use of filament-wound GFRP tubes for shorter BRB and pultruded GFRP tubes for longer BRBs.

### 4.2. Assembled BRBs

**Figure 16:**
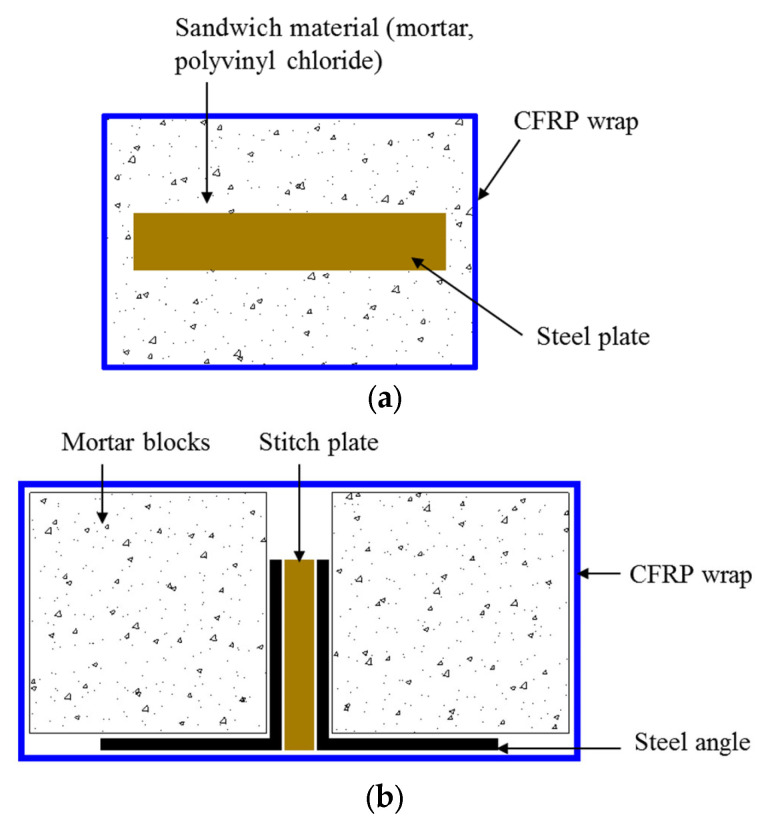

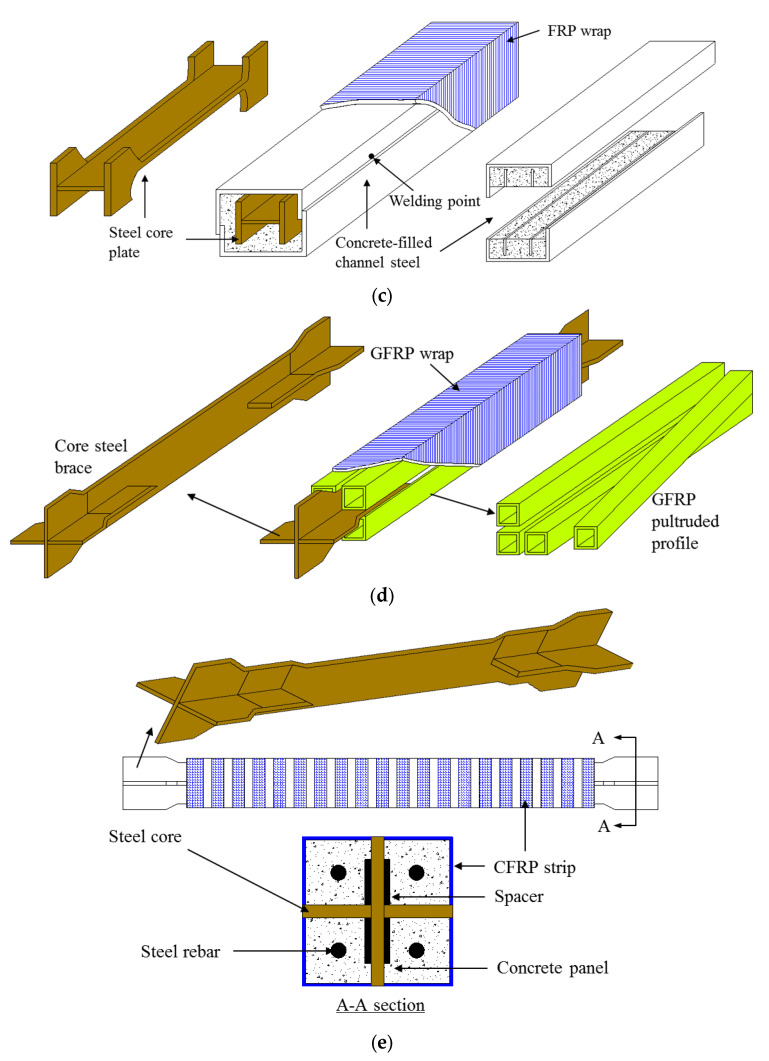
Assembled BRB wrapped by FRPs. (**a**) Ekiz and El-Tawil [64]. (**b**) El-Tawil and Ekiz [65]. (**c**) Jia et al. [67]. (**d**) Deng et al. [66]. (**e**) Bashiri and Toufigh [68].

Assembled BRBs usually involve FRP wraps. They can be assembled or disassembled by wrapping or cutting the external FRP wraps, respectively, making repair or inspection easier. Research related to this form of BRBs has been extensively carried out [64,65,66,67,68]. Ekiz and El-Tawil [64] conducted an experimental and computational study to investigate the monotonic compressive behavior of small-scale BRBs with mortar or polyvinyl chloride (PVC) blocks as filling material and externally bonded CFRP sheets in the longitudinal and transverse directions (Figure 16a). Assembled BRBs are susceptible to stress concentration, so increasing the number of longitudinal CFRP layers can effectively prevent the premature rupture of the FRP wrap and improve the load-carrying capacity of the BRBs. Moreover, Ekiz and El-Taiwil [64] studied the effects of bonds. They demonstrated that the presence of the bond between the steel plate and the filling materials had an adverse effect on compressive ductility, as overall buckling appeared to occur earlier, but it was beneficial to inhibit the premature buckling of the FRP wrap.

The cyclic behavior of assembled BRBs with FRP wrapping has attracted the attention of some researchers. El-Taiwil and Ekiz [65], Deng et al. [66], and Jia et al. [67] carried out reversed cyclic axial loading tests on different configurations of large-scale assembled BRBs. As shown in Figure 16b, El-Taiwil and Ekiz [65] proposed a new strengthening technique in which a core composed of pre-fabricated mortar blocks was attached to double steel angle sections and the entire system was wrapped with CFRP sheets in the longitudinal (main direction) and transverse fibers directions. They found that buckling restrained response could reach up to 2% inter-story drift. In the study of Jia et al. [67], the proposed BRBs comprised three components: a steel core plate, a pair of concrete-filled channel steel, and wrapped FRP clothes (Figure 16c). In addition, Deng et al. [66] designed a novel hybrid BRB in which four GFRP-pultruded tubes were tied to the core steel brace and cruciform cross section together and externally wrapped with GFRP layers (Figure 16d). On the other hand, Bashiri and Toufigh [68] utilized FRP partial wrapping strengthening schemes in BRBs. In their study, a pair of BRBs with a dog-bone-shaped steel core restrained with RC panels and wrapped with CFRP strips (Figure 16e) was placed into a half-scale steel frame, and the frame was tested under cyclic loading to assess the cyclic behavior of the proposed BRBs. It was demonstrated that the partial confinement of CFRP strips could provide adequate strength and stiffness for the BRBs to prevent buckling of the steel core.

## 5. Conclusions

Previous studies have indicated that FCSRC structural members are promising. This paper has presented a state-of-the-art review of FCSRC structural members in strengthening existing structures/constructing new structures and buckling restrained braces. Based on the literature review, several key conclusions can be drawn:

(1) Using FRP confining devices and filling materials (the concept of FCSRC systems) to strengthen existing (corroded and buckled) steel columns is feasible: the load-carrying capacity of reinforced steel columns can be restored or even significantly increased to some extent due to the dual restraint of concrete and FRP tubes. In this case, in addition to the FRP-wrapping-based wet layup process, the split-tube construction process is also recommended.
(2) FCSRCs have been developed into significant structural elements in new structures (e.g., hybrid columns or beams). The FRP confinement and composite action between the three components (i.e., steel, concrete, and FRP) generally result in the superior structural performance of FCSRC structural members.
(3) Previous studies have primarily focused on the behavior of circular, square, and rectangular FCSRC columns subjected to concentric or eccentric compression. The investigated parameters included the cross-sectional shapes, the strength grades of the steel or the concrete, the slenderness ratio, and the load eccentricity.
(4) In most cases, the buckling of inner steel section (especially overall buckling) can be effectively delayed or prevented by the surrounding concrete and the external FRP confining tube no matter its configuration, so the post-yield strength of steel can be fully exploited, further indicating the validation of the FCSRC system.
(5) In addition to the FRP full wrapping strengthening, FRP partial wrapping strengthening has been used in FCSRC columns, and relevant research has indicated the feasibility of using FRP strips to confine concrete-encased steel columns.
(6) New types of materials have been adopted in FCSRC columns. For instance, PET FCSRC columns exhibit much better deformation capacity than FCSRC columns made of conventional FRPs. PET FCSRC columns can fully exploit the strength of high-strength materials including high-strength steel and high-strength concrete.
(7) Pre-stress could eliminate stress lag, and the strength of expansive concrete-based FCSRC columns is higher than that of FCSRC columns with ordinary concrete.
(8) A number of models have been proposed for the load capacity and ultimate axial strain of FCSRC columns. These models are classified into three types based on the estimation of the contributions of three different components in FCSRC columns (i.e., FRP, concrete, and steel). However, these models have never considered the additional confinement from the steel section. The accuracy of these models also requires further evaluation.
(9) Currently, research on FCSRC beams is rather limited. A previous study indicated the excellent flexural performance of FCSRC beams.
(10) The concept of FCSRC systems has been applied to buckling restrained braces. Previous studies on FCSRC BRBs have focused on behavior under the axial compression or reversed cyclic loading, and they have demonstrated the feasibility of FRP strengthening technique for BRBs.

## 6. Future Opportunities

In order to facilitate the wide application of FCSRC systems in structural applications, it is necessary to gain an in-depth understanding of the structural performance of FCSRC systems subjected to various forms of loading. The gaps in knowledge and future research opportunities on FCSRC systems are identified and discussed below:

(1) The effects of full and partial encasement of steel sections in surrounding concrete need to be explored.
(2) The long-term structural performance of FCSRC structural members under extreme conditions (e.g., seismic, blast, impact, and aggressive environmental attacks) needs to be further explored.
(3) Future efforts can focus on the development of FCSRC structural members made of high-performance materials.
(4) The buckling behavior of steel sections may counteract the strength enhancement caused by their confinement; however, most models still assume that the steel is an idealized elastic–perfectly plastic material. Thus, it requires further investigation regarding the two aspects, and respective design standards need to be established.
(5) Research on FCSRC beams is rather limited. The fatigue performance of FCSRC beams needs to be explored.
(6) The bond behavior between the steel section and concrete in an FCSRC member needs to be understood. The combined use of bolt connections can result in full composite action. The interfacial bond between FRP and the concrete could be enhanced by using a proper device (such as a rough surface of the FRP with resin ribs or sand-coating).
(7) The fire resistance of exterior FRP coatings is very important when FCSRC structural members are used in residential premises. It is necessary to adopt an effective approach (such as a fire-redundant coatings, as per GB 50608 [99]) to improve the fire performance of FRP materials.
(8) It is necessary to develop new types of FCSRC BRBs that are more efficient and inexpensive.
(9) FCSRCs are generally used as columns, beams, and buckling restrained braces in high-rise buildings or infrastructures. FCSRCs have been less used in spatial structures to date, which deserves further investigation.
(10) Future work should address the issue of FRP layer protection against mechanical damage.

## Figures and Tables

**Table 1:** Summary of studies on FCSRC columns under axial compression.

Reference	No. of Specimens	Loading Pattern	Cross Section	FRP Type	Concrete Type	Steel Shape	Steel Type	Investigated Parameters
Liu et al. [32]	7	Concentric	Circular	GFRP	Non-expansive and expansive light-weight concrete	I-section	NSS	N.A.
Cao et al. [39]	24	Concentric	Square	CFRP	Ordinary concrete and expansive concrete	I-section	NSS	Pre-stress, layers of CFRP, dimensions of section steel
Cao et al. [40]	24	Concentric	Circular	CFRP	Ordinary concrete, expansive concrete	I-section	NSS	Pre-stress, layers of CFRP, dimensions of section steel
Chen et al. [41]	22	Concentric	Circular	GFRP	Normal-strength concrete, High-strength concrete	I-section, Cruciform section	NSS	Concrete strength, Steel section shape, FRP tube thickness
Karagah et al. [55]	14	Concentric	Circular	GFRP, CFRP	Grout	I-section,	NSS	Different degrees of corrosion, longitudinal steel reinforcing bars, headed-stub anchors, FRP jacket configuration
He and Chen [54]	27	Concentric	Circular	GFRP	Ordinary concrete	Channel steel	NSS	Steel ratio, concrete compressive strength, diameter–thickness ratio of GFRP tube
Huang et al. [42]	12	Concentric	Square	GFRP	Ordinary concrete	Cruciform section	NSS	FRP tube thickness
Huang et al. [43]	12	Concentric	Circular	PET FRP	Ordinary concrete	I-section	NSS	FRP tube thickness
Huang et al. [44]	16	Concentric, eccentric	Square	GFRP	Ordinary concrete	Cruciform section	NSS	Slenderness ratio, load eccentricity, FRP tube thickness
Huang et al. [45]	24	Concentric	Square	GFRP	Ordinary concrete	Cruciform section	NSS	Flange width, flange thickness, web thickness, FRP tube thickness
Karimi et al. [34]	7	Concentric	Rectangular	GFRP, CFRP	Ordinary concrete	I-section (partially encased by concrete)	NSS	CFRP thickness, corner radius
Karimi et al. [37]	7	Concentric	Circular	GFRP	Ordinary concrete, expansive concrete	I-section	NSS	Type of GFRP tube, shrinkage-reducing agent
Karimi et al. [38]	9	Concentric	Circular	GFRP	Ordinary concrete	I-section	NSS	Slenderness ratio
Karimi et al. [35]	9	Concentric	Rectangular	GFRP, CFRP	Ordinary concrete	I-section (partially encased by concrete)	NSS	Slenderness ratio
Kaya et al. [52]	13	Concentric	Circular	GFRP	Expansive concrete	I-section	NSS	Number of layers of GFRP jacket, the presence and diameter of internal longitudinal steel reinforcing bars
Liang et al. [46]	14	Concentric	Square	CFRP	Ordinary concrete	I-section	NSS	Number of CFRP strip layers, net spacing of CFRP strip, slenderness ratio
Linde et al. [36]	18	Concentric	Circular	GFRP, CFRP	Ordinary concrete, shrinkage-reducing admixture (SRA) concrete	I-section	NSS	Adding confined concrete, using a split-tube system, adding shrinkage-reducing admixtures
Ozbakkaloglu and Fanggi [53]	2	Concentric	Circular	CFRP	High-strength concrete	I-section	NSS	N.A.
Ren et al. [47]	41	Concentric	Circular	GFRP	High-strength concrete	H-section, cruciform section	NSS	FRP tube thickness, encased steel shape, ratio to the area of steel plus concrete core
Ren et al. [48]	9	Eccentric	Circular	GFRP	Recycled aggregate concrete (RCA), natural aggregate concrete	I-section	NSS	Replacement ratio of RCA, FRP tube thickness, load eccentricity, slenderness ratio
Xie et al. [56]	30	Concentric	Circular	GFRP	Ordinary concrete	I-section	NSS	Steel ratio, thickness of GFRP tube, concrete strength
Xiong et al. [49]	24	Concentric	Circular	GFRP	Recycled aggregate concrete (RCA), natural aggregate concrete	Cruciform section	NSS	Replacement ratio of RCA, FRP tube thickness
Yu et al. [50]	14	Concentric, eccentric	Square, Circular	GFRP	Ordinary concrete	I-section	NSS	Sectional configuration, FRP tube thickness, loading scheme, load eccentricity
Yu et al. [51]	13	Concentric, eccentric	Circular	GFRP	Ordinary concrete	Steel plate	NSS, HSS	Configuration of steel plates, steel grade, thickness of steel plates, FRP tube thickness, loading scheme

Note: GFRP—Glass FRP; CFRP—Carbon FRP; PET FRP—Polyethylene terephthalate FRP; NSS—Normal-strength steel; HSS—High-strength steel (refers to the steel with a yield stress not lower than 450 MPa); N.A.—No applicable.

**Table 2:** Summary of studies on FCSRC BRBs.

Reference	No. of Specimens	Loading Pattern	Cross Section	FRP Type	Filling Material	Steel Shape	Investigated Parameters
Ekiz and El-Tawil [64]	22	Concentric compression	Rectangular	CFRP wrap	Mortar, polyvinyl chloride (PVC)	Steel plate	Number of longitudinal CFRP layers, core thickness, bond between CFRP layers and the core, bond between the core and the inner steel member, strength of transverse sheets at the member ends
El-Tawil and Ekiz [65]	7	Reversed cyclic loading	Rectangular	CFRP wrap	Mortar blocks	Steel plate	End boundary condition, number of layers of CFRP sheets, mortar cross section, use of extra stitch plates, steel/CFRP bond
Feng et al. [58]	14	Concentric compression	Circular	Pultruded GFRP tube	RGM-high strength non-shrinkage grout	L-shaped steel	Slenderness ratio, the confinement detail, the filled materials, the end connection
Feng et al. [59]	18	Concentric compression	Circular	Pultruded GFRP tube	Mortar	Cruciform section, I-section, round tube, square tube	Cross section of core steel, slenderness, FRP fabric layers wrapped at the ends of specimens
Deng et al. [66]	1	Reversed cyclic loading	Square	Pultruded GFRP tube and GFRP wrap	High-strength non-shrinkage mortar	Cruciform section	Thickness and wrapping angle of the GFRP wraps
Jia et al. [67]	8	Reversed cyclic loading	Rectangular	CFRP, basalt FRP wrap	C30 concrete	Steel plate	Length of the steel core plate, FRP type, loading protocols
Sun et al. [63]	14	Reversed cyclic loading	Circular	Pultruded GFRP tube, filament-wound GFRP tube	Fine aggregate concrete	Steel plate, I-section	Constraint ratio of BRBs, restraining component size, specimen length, the thickness and the type of the external GFRP tubes
MacEachern and Sadeghian [62]	36	Concentric compression	Circular	Manually prefabricated GFRP tube	Self-consolidating grout	Hot-rolled steel bar	Three different FRP shell lengths, three different outer shell diameters
Bashiri and Toufigh [65]	2	Reversed cyclic loading	Square	CFRP (partial) wrap	Concrete	Dog-bone-shaped steel core	N.A.

**Table 3:** Typical properties of various fibers in FRPs (data from manufacturer).

Fiber Type	Tensile Strength (MPa)	Modulus of Elasticity (GPa)	Ultimate Tensile Strain
Carbon	3790	242	1.55
Glass	1720	72	2.4
Basalt	1000	50	2.24
Aramid	2060	118	1.8
PET	740	10 ± 1	>7.0
PEN	790	15 ± 2	>5.0
PA	1760	40	6~9

**Table 4:** Summary of models proposed for FCSRC columns/the respective confined concrete.

Reference	Model
Type I: Considers the contributions of unconfined concrete and profile steel in the load capacity of FCSRC columns (i.e., the nominal load capacity $P_{co}$).	
Cao et al. [39]	$P_{co}=f_{co}^{\prime }A_{c}+f_{y}A_{s}$ $f_{cu}^{\prime }=\frac{P_{cu}-f_{y}A_{s}}{A_{c}}\;$ (Test) Proposed confined concrete model: $\frac{f_{cu}^{\prime }}{f_{co}^{\prime }}=1+3.3k_{s}\frac{f_{l}}{f_{co}^{\prime }}+3.5k_{s}\frac{f_{l}^{*}}{f_{co}^{\prime }}$ $\frac{\varepsilon_{cu}}{\varepsilon_{co}}=1.75+12k_{s}\frac{f_{l}}{f_{co}^{\prime }}{\left(\frac{\varepsilon_{h,rup}}{\varepsilon_{co}}\right)}^{0.45}+17.5k_{s}\frac{f_{l}^{*}}{f_{co}^{\prime }}$ $k_{s}=1-\frac{2}{3}\frac{{\left(1-2\frac{r}{b}\right)}^{2}}{1-\left(4-\pi \right){\left(\frac{r}{b}\right)}^{2}}\approx 0.75$ $f_{l}=\frac{E_{f\;}t_{f}\varepsilon_{f,rup}}{D\;}$$\;(D=\sqrt{2}b-2r\left(\sqrt{2}-1\right)$)
Type II: Considers the contributions of confined concrete and profile steel in the load capacity $P_{cc}$ of FCSRC columns.	
Chen et al. [41], Xiong et al. [49]	The superimposed load capacity $P_{o}$: $P_{o}=\frac{A_{g}-A_{s}}{A_{g}}P_{CFFT}+P_{s}\;\left(P_{s}=f_{y}A_{s}\right)$ $P_{cu}\;\left(\mathrm{Test}\right)>P_{o}$ for FCSRCs with I-section steel $P_{cu}\;\left(\mathrm{Test}\right)<P_{o}$ for FCSRCs with cruciform section steel
Liang et al. [46]	$P_{cc}=\varphi \left(0.85f_{co}^{\prime }A_{c}+2k_{g}f_{l}A_{c}+f_{y}A_{s}\right)$ $k_{g}={\left(1-\frac{s_{f}}{2d_{min}}\right)}^{2}$ $f_{l}=\frac{2f_{f}t_{f}}{\sqrt{h^{2}+b^{2}}}$ $d_{min}=min\left\{h,b\right\}$
Huang et al. [45]	$P_{cc}=f_{cu}^{\prime }A_{c}+f_{y}A_{s}$ Confined concrete model of Teng et al. [92]: $\frac{f_{cu}^{\prime }}{f_{co}^{\prime }}=\left\{\begin{array}{cc} 1+3.5\left(\rho_{K}-0.01\right)\rho_{\varepsilon } & if\;\rho_{K}\geq 0.01\\ 1 & if\;\rho_{K}\leq 0.01 \end{array}\right.$ $\varepsilon_{cu}=\varepsilon_{co}[1.75+1.65\rho_{K}{}^{0.8}\rho_{\varepsilon }{}^{1.45}$] $\rho_{K}=\frac{2E_{f\;}t_{f}}{\left(f_{co}^{\prime }/\varepsilon_{co}\right)D\;}$ $\rho_{\varepsilon }=\frac{\varepsilon_{f,rup}}{\varepsilon_{co}\;}$
Ren et al. [48] *	The ultimate load-carrying capacity of the slender FCSRC columns under eccentric compression: $N_{u}=\frac{1-0.55\bar{\lambda }}{1+0.45\bar{\lambda }}\frac{1-\frac{0.7e}{D}}{1+\frac{2.7e}{D}}\left(f_{cu}^{\prime }A_{c}+f_{y}A_{s}\right)$ $\bar{\lambda }=\sqrt{\frac{f_{cu}^{\prime }A_{c}+f_{y}A_{s}}{\pi^{2}{\left(EI\right)}_{eff,I}/L_{eff}^{2}}}$ ${\left(EI\right)}_{eff,I}=I_{eq}E_{c}$ $I_{eq}=\left(\frac{E_{s}}{E_{c}}-1\right)I_{s,m}+\frac{\pi }{64}{\left(D+2t_{f}\frac{E_{a}}{E_{c}}\right)}^{4}$ Confined concrete model of Teng et al. [92]: $\frac{f_{cu}^{\prime }}{f_{co}^{\prime }}=\left\{\begin{array}{cc} 1+3.5\left(\rho_{K}-0.01\right)\rho_{\varepsilon } & if\;\rho_{K}\geq 0.01\\ 1 & if\;\rho_{K}\leq 0.01 \end{array}\right.$ $\rho_{K}=\frac{2E_{f\;}t_{f}}{\left(f_{co}^{\prime }/\varepsilon_{co}\right)D\;}$ $\rho_{\varepsilon }=\frac{\varepsilon_{f,rup}}{\varepsilon_{co}\;}$
He and Chen [54]	$P_{cc}=\theta \left(0.8f_{co,cube}^{\prime }A_{c}+f_{y}A_{s}\right)$ $\theta =\left(46.48\alpha^{-1}+1.72\right)\times 0.3^{\beta }\times 0.9^{\gamma }$ $\alpha =D/t_{f}$ (diameter–thickness ratio of GFRP tube) $\beta =A_{s}/A_{g}$ (steel ratio) $\gamma =f_{co,cube}^{\prime }/15$
Type III: Considers the contributions of three parts (i.e., confined concrete, profile steel, and FRP tube) in the load capacity $P_{cc}$ of FCSRC columns.	
Karimi et al. [37,90] *	$P_{cc1}=f_{cc}^{\prime }A_{c}+f_{su}A_{s}+\sigma_{f,au}A_{f}$ Confined concrete model of Lam and Teng [93]: $f_{cc}^{\prime }=f_{co}^{\prime }+3.3\frac{2\sigma_{f,lu}t_{f}}{D}$ $\varepsilon_{cu}=\varepsilon_{co}\left[1.75+12\frac{2\sigma_{f,lu}}{Df_{co}^{\prime }}{\left(\frac{\varepsilon_{f,lu}}{\varepsilon_{co}}\right)}^{0.45}\right]$ The GFRP tube is under a biaxial state of stress: $\sigma_{f,au}=\frac{E_{fa}}{1-\nu_{al}\nu_{la}}\varepsilon_{f,au}+\frac{\nu_{al}E_{fa}}{1-\nu_{al}\nu_{la}}\varepsilon_{f,lu}$ $\sigma_{f,lu}=\frac{\nu_{la}E_{fl}}{1-\nu_{al}\nu_{la}}\varepsilon_{f,au}+\frac{E_{fl}}{1-\nu_{al}\nu_{la}}\varepsilon_{f,lu}$ $(\varepsilon_{cu}=\varepsilon_{f,au}$) Tsai–Wu failure criterion: $\left(\frac{1}{S_{l,t}}-\frac{1}{S_{l,c}}\right)\sigma_{f,lu}+\left(\frac{1}{S_{a,t}}-\frac{1}{S_{a,c}}\right)\sigma_{f,au}+\frac{1}{S_{l,t}S_{l,c}}\sigma_{f,lu}^{2}\;+\frac{1}{S_{a,t}S_{a,c}}\sigma_{f,au}^{2}-\frac{1}{\sqrt{S_{a,c}S_{a,t}S_{l,c}S_{l,t}}}\sigma_{f,lu}\sigma_{f,au}=1$ The ultimate load-carrying capacity of the slender FCSRC columns under axial compression [90]: $P_{cc2}=P_{cc1}\left(1+\lambda^{2}\right)$ $\lambda =\sqrt{\frac{P_{cc1}}{P_{cE}}}=\sqrt{\frac{P_{cc1}{\left(kL\right)}^{2}}{\pi^{2}EI}}$$EI=E_{s}I_{s}+E_{c}I_{c}+E_{fa}I_{f}$ $(E_{c}=E_{2}=\frac{f_{cc}^{\prime }-f_{co}^{\prime }}{\varepsilon_{cu}}$)
Ren et al. [47]	$P_{cc}=\sigma_{f,au}A_{f}+f_{cu}^{\prime }A_{c}+P_{s}\;$ $P_{s}=\left\{\begin{array}{cc} E_{s}\varepsilon_{cu}A_{s} & if\;E_{s}\varepsilon_{cu}\leq f_{y}\\ f_{y} & if\;E_{s}\varepsilon_{cu}>f_{y} \end{array}\right.$ A path-dependent stress–strain model of concrete confined with FRP: $\varepsilon_{cu}=0.578{\left(\frac{f_{co}^{\prime }}{30}\right)}^{m}\left\{\varepsilon_{co}{\left[1+0.75\left(\frac{-\varepsilon_{f,rup}}{\varepsilon_{co}}\right)\right]}^{0.7}-\varepsilon_{co}exp\left[7\left(\frac{\varepsilon_{f,rup}}{\varepsilon_{co}}\right)\right]+0.07{\left(-\varepsilon_{f,rup}\right)}^{0.7}\left[1+26.8\left(\frac{f_{l}}{f_{co}^{\prime }}\right)\right]\right\}$ $m=\left\{\begin{array}{cc} 0 & if\;f_{co}^{\prime }\leq 30\\ -0.05 & if\;f_{co}^{\prime }>30 \end{array}\right.$ $\frac{f_{cu}^{\prime }}{f_{cc}^{\prime *}}=\frac{A\left(\varepsilon_{cu}/\varepsilon_{cc}^{*}\right)+B{\left(\varepsilon_{cu}/\varepsilon_{cc}^{*}\right)}^{2}}{1+\left(A-2\right)\left(\varepsilon_{cu}/\varepsilon_{cc}^{*}\right)+\left(B+1\right){\left(\varepsilon_{cu}/\varepsilon_{cc}^{*}\right)}^{2}}$ $\frac{f_{cc}^{\prime *}}{f_{co}^{\prime }}=1+3.5\left(\frac{f_{l}}{f_{co}^{\prime }}\right)$ $\varepsilon_{cc}^{*}=\varepsilon_{co}\left[1+\left(17.0-0.06f_{co}^{\prime }\right)\frac{f_{l}}{f_{co}^{\prime }}\right]$ (A and B are curve shape parameters [94]) $f_{l}=-\frac{2\sigma_{f,lu}t_{f}}{D}$ The GFRP tube is under a biaxial state of stress: $\sigma_{f,au}=\frac{E_{fa}}{1-\nu_{al}\nu_{la}}\varepsilon_{f,au}+\frac{\nu_{al}E_{fa}}{1-\nu_{al}\nu_{la}}\varepsilon_{f,lu}$ $\sigma_{f,lu}=\frac{\nu_{la}E_{fl}}{1-\nu_{al}\nu_{la}}\varepsilon_{f,au}+\frac{E_{fl}}{1-\nu_{al}\nu_{la}}\varepsilon_{f,lu}$ $(\varepsilon_{cu}=\varepsilon_{f,au}$)
Xie et al. [56]	$P_{cc}=\left[\frac{f_{y}A_{s}+f_{co}^{\prime }A_{c}}{A_{s}+A_{c}}+5.9{\left(2\frac{\sigma_{f}t_{f}}{D}\right)}^{0.689}\right]A_{c}+\sigma_{fu}A_{f}$ $f_{co}^{\prime }=0.8f_{co,cube}^{\prime }$

Note: *—Slender columns; $A_{c}$—Cross-sectional area of the concrete; $A_{f}$—Cross-sectional area of the FRP tube; $A_{g}$—Gross-sectional area of the column; $A_{s}$—Cross-sectional area of the steel section; b and r—Side length and corner radius of the square column, respectively; D—Outer diameter of the FRP tube; e/D—Load eccentricity ratio; $E_{fa\;}$—Axial compressive modulus of the FRP tube; $E_{f\;}$(or $E_{fl\;}$)—Hoop tensile modulus of the FRP tube; $E_{s}$—Elastic modulus of the steel; $f_{cc}^{\prime }$—Compressive strength of confined concrete; $f_{co}^{\prime }$—Compressive strength of unconfined concrete; $f_{co,cube}^{\prime }$—Compressive strength of concrete cube; $f_{cu}^{\prime }$—Ultimate axial stress of confined concrete; $f_{f}$—Tensile strength of FRPs; $f_{l}$—Confining stress generated from the FRP; $f_{l}^{*}$—Equivalent pre-stress generated from expansive concrete; $f_{su}$—Ultimate tensile strength of the steel; $f_{y}$—Yield strength of the steel; h—Height of rectangular column; $I_{s}$, $I_{c}$, and $I_{sf}$—Moment of inertia of the steel, the concrete, and the FRP tube, respectively; $I_{s,m}$—Moment of inertia of the steel section with respect to its major axis; k—Effective length factor; L—Unbraced column length; $L_{eff}$—Column effective length; $P_{CFFT}$—Load capacity of the CFFT specimen; $P_{cu}$—Ultimate axial load capacity of the tested specimens; $S_{a,t}$ and $S_{a,c}$—Tensile and compressive strength values in the axial direction, respectively; $S_{l,t}$ and $S_{l,c}$—Tensile and compressive strength of the FRP tube in the hoop direction, respectively; $s_{f}$—Pitch of FRP strips (center-to-center); $t_{f}$—Thickness of the FRP; $\varepsilon_{co}$—Axial strain of unconfined concrete at peaks; $\varepsilon_{cu}$—Ultimate axial strain of confined concrete; $\varepsilon_{h,rup}$—Actual hoop rupture strain of the FRP; $\varepsilon_{f,au}$ and $\varepsilon_{f,lu}$—Ultimate axial and hoop strains measured on the FRP tube; $v_{al}$ (or $v_{la}$—Poisson’s ratio for the hoop (or axial) strain when subjected to axial (or hoop) stress; $\sigma_{f,au}$ and $\sigma_{f,lu}$—Ultimate axial and hoop stresses measured on the FRP tube, respectively; $\sigma_{f}$—Hoop tensile strength of the FRP tube; $\sigma_{fu}$—Compression strength of the hollow FRP tube; φ—Buckling factor of column as per GB50017-2017 (2017) [95].

## Data Availability

The data presented in this study are available on request from the corresponding author.
